# In-Plane Impact Response of Re-Entrant Auxetic Honeycombs with Spatially Graded Material Strength

**DOI:** 10.3390/ma19214674

**Published:** 2026-11-03

**Authors:** Mehmet Seha Tatlıer

**Affiliations:** Mechanical Engineering Department, Osmaniye Korkut Ata University, 80000 Osmaniye, Turkey; sehatatlier@osmaniye.edu.tr

**Keywords:** re-entrant auxetic honeycomb, functionally graded material, yield-strength grading, dynamic crushing, in-plane impact, energy absorption, crush force efficiency

## Abstract

Re-entrant auxetic honeycombs have attracted considerable interest as lightweight energy-absorbing structures because of their distinctive deformation mechanisms and favorable crushing characteristics. This study investigates the dynamic in-plane crushing response of isotropic and functionally graded re-entrant honeycombs, emphasizing material-property grading rather than conventional geometric grading. Cellular geometry, relative density, and structural mass were maintained identically, while grading was introduced exclusively through continuous variation in parent-material yield strength along the crushing direction. A rate-independent elastic–perfectly plastic model was employed at impact velocities of 50, 75, and 100 m/s, and the numerical response was assessed against an established analytical prediction of dynamic crushing resistance. The results show that strength grading influences collapse localization, force transmission, and progressive cellular compaction. Compared with the isotropic configuration, the graded structures exhibit reduced maximum crushing forces while maintaining or improving mean crushing resistance, energy absorption, specific energy absorption, and crush force efficiency. The effects depend on impact velocity and reflect interactions among structural inertia, collapse progression, and spatially distributed plastic resistance. A relatively compliant impact-side region facilitates controlled deformation initiation, while progressively stronger regions sustain resistance during subsequent collapse. Overall, strength grading improves the balance between peak-load reduction and energy absorption without changing cellular architecture or structural mass.

## 1. Introduction

Auxetic materials are characterized by a negative Poisson’s ratio: under tensile loading they may expand, rather than contract, in a transverse direction. In architected solids this response often results from internal deformation mechanisms rather than an intrinsically auxetic parent material. Gibson et al. provided an early analysis of two-dimensional cellular materials, including inward-inclined (re-entrant) configurations, while Lakes demonstrated negative-Poisson-ratio foams experimentally in 1987 [1,2]. Subsequent assessments established the breadth of auxetic lattice mechanisms and their dependence on cell geometry [3]. Re-entrant honeycombs are therefore attractive for lightweight impact absorbers, but the term auxetic should not be taken to imply a negative Poisson’s ratio for every loading direction or throughout large deformation. Their effective response is anisotropic and can vary with orientation, strain and geometric proportions [4,5,6].

The inward-pointing, non-convex geometry of a re-entrant cell permits its inclined ligaments to rotate and bend, producing lateral motion that differs from that of a conventional convex hexagon. Non-convexity is also relevant at a distinct, molecular scale: simulations of interacting hard cyclic hexamers identified thermodynamically stable phases with negative Poisson’s ratios, and subsequent analytical work described a stable isotropic auxetic model [7,8]. Studies of cyclic tetramers further distinguish fully auxetic, partially auxetic and non-auxetic phases [9]. These molecular phases are not mechanically equivalent to fabricated honeycombs; they illustrate that auxeticity may emerge from collective rearrangement at different length scales. For the present re-entrant lattice, partially auxetic is a more precise description than claiming an omnidirectional negative Poisson’s ratio. Other non-conventional cellular morphologies, including irregular cosinecomb panels, demonstrate that auxetic mechanisms are not restricted to straight-rib re-entrant hexagons [10].

The crushing performance of a honeycomb depends not only on its effective Poisson’s ratio but also on ligament buckling, plastic-hinge formation, contact and progressive densification. Foundational investigations of conventional honeycombs clarified how deformation fronts and inertia influence in-plane crushing under dynamic loads [11,12,13,14]. Re-entrant honeycombs introduce additional geometrical sensitivity because inclined walls may bend, rotate and interact in highly localized zones. Dynamic numerical and theoretical investigations have demonstrated changes in deformation patterns with cell-wall angle, loading direction and impact speed [15,16,17,18]. In particular, the analytical treatment of Hu et al. separates a plastic-collapse contribution from velocity-related inertial resistance and provides a useful reference for interpreting the dynamic response of an otherwise uniform re-entrant structure [15]. Such results motivate examining how plastic resistance is distributed spatially rather than treating the lattice as a uniform crushing medium.

Numerous strategies have sought to improve cellular crashworthiness through geometric redesign. Modified re-entrant cells, hierarchical layouts and alternative auxetic topologies can alter collapse stability and energy absorption [19,20,21,22]. Bio-inspired, combined and sinusoidally modified configurations further enlarge the available design space [23,24,25]. The development of curved, circular, hybrid and multi-plateau architectures illustrates the capacity to guide successive collapse events through cell morphology [26,27,28,29,30,31]. More recent analytical–numerical comparisons likewise emphasize the coupling between re-entrant kinematics and dynamic deformation [32]. Collectively, these investigations establish topology as a powerful design parameter, but they do not by themselves isolate the effects of a spatial change in the mechanical properties of the constituent material.

Functionally graded cellular structures offer a different route for tailoring resistance along the crushing path. Many studies implement geometric grading by varying cell size, ligament thickness, cell angle, structural hierarchy or relative density. Density-gradient and piecewise-linear honeycombs demonstrated the influence of non-uniform cellular architectures on progressive crushing [33,34]. Studies of graded hierarchical and angular-gradient honeycombs subsequently expanded these geometric approaches [35,36,37]. Such designs can improve load management, yet changes in local architecture may also change stiffness, relative density and, unless compensated elsewhere, total mass. Consequently, an apparent benefit in absorbed energy or peak force can originate from several coupled design changes. This complicates attempts to determine whether the improvement derives from grading the parent material itself or from altering the distribution of structural material.

Grading has also been investigated within re-entrant families. Fabricated graded re-entrant circular systems, graded auxetic re-entrant honeycombs and additively manufactured non-homogeneous re-entrant specimens provide evidence that spatial variation can influence crushing modes [38,39,40]. However, geometrical and material heterogeneity must be distinguished explicitly. In material-property grading, the cellular topology, ligament dimensions, relative density and mass are kept fixed, while selected constitutive parameters vary with position. If only the parent-material yield strength is varied, the local onset of plastic deformation changes without introducing a new stiffness distribution from geometry or adding material. This establishes a controlled comparison with a uniform-strength reference. It is particularly relevant when an impact absorber is subject to strict packaging and mass constraints and when the designer seeks to redistribute plastic dissipation rather than maximize resistance indiscriminately.

For re-entrant networks, a yield-strength gradient oriented along the crushing direction offers a physically interpretable mechanism. A weaker region adjacent to the impact surface may initiate plastic-hinge formation at relatively low resistance, while stronger regions activated later may help sustain the mean crushing load. In contrast, a uniformly weakened lattice could reduce both maximum and sustained resistance. A graded response must also be assessed dynamically: stress-wave transients, structural inertia, propagation of the collapse front, ligament contacts and terminal densification can affect the location and magnitude of peak force. Studies of dynamic crushing and theoretical strength prediction demonstrate the importance of separating velocity-dependent structural effects from changes in parent-material strength [5,6,15,31]. Whether yield-strength grading can reduce peak load while preserving energy absorption at identical structural mass therefore remains a distinct research question, not a result that follows automatically from successful geometric grading.

The present study addresses this question using finite-element analysis (FEA) of re-entrant honeycombs subjected to in-plane impact at 50, 75 and 100 m/s. A uniform-strength reference, denoted ISO, is compared with a functionally graded material (FGM) configuration in which the yield strength increases continuously from the impacted boundary toward the fixed boundary. The reference and graded models use identical cells, ligament thicknesses, elastic constants, density, relative density and total mass. A rate-independent elastic–perfectly plastic constitutive description is adopted so that changes with impact velocity are associated with structural dynamics and deformation progression rather than prescribed intrinsic strain-rate strengthening. This configuration separates material-strength redistribution from mass or geometric grading and allows its effects to be evaluated without changing the underlying re-entrant architecture.

The analysis focuses on force–displacement evolution, maximum and mean crushing forces, absorbed energy, specific energy absorption (SEA) and crush force efficiency (CFE), together with the progression of localized collapse. Its principal contribution is an ISO–FGM comparison in which the spatial distribution of yield strength is the sole intentionally varied structural parameter. The emphasis is on the balance between transmitted peak force and sustained energy-dissipation capacity across impact velocities, rather than on obtaining a higher strength through added material. Because the investigated architecture is direction dependent, the findings should be interpreted for the specified loading orientation; comparing equivalently graded convex hexagonal honeycombs would be a valuable extension but is outside the present numerical study. The results provide a basis for evaluating whether material-property grading can control dynamic crushing resistance without sacrificing the geometric and mass advantages of the original re-entrant honeycomb.

## 2. Materials and Methods

### 2.1. Numerical Modelling

**Figure 1:**
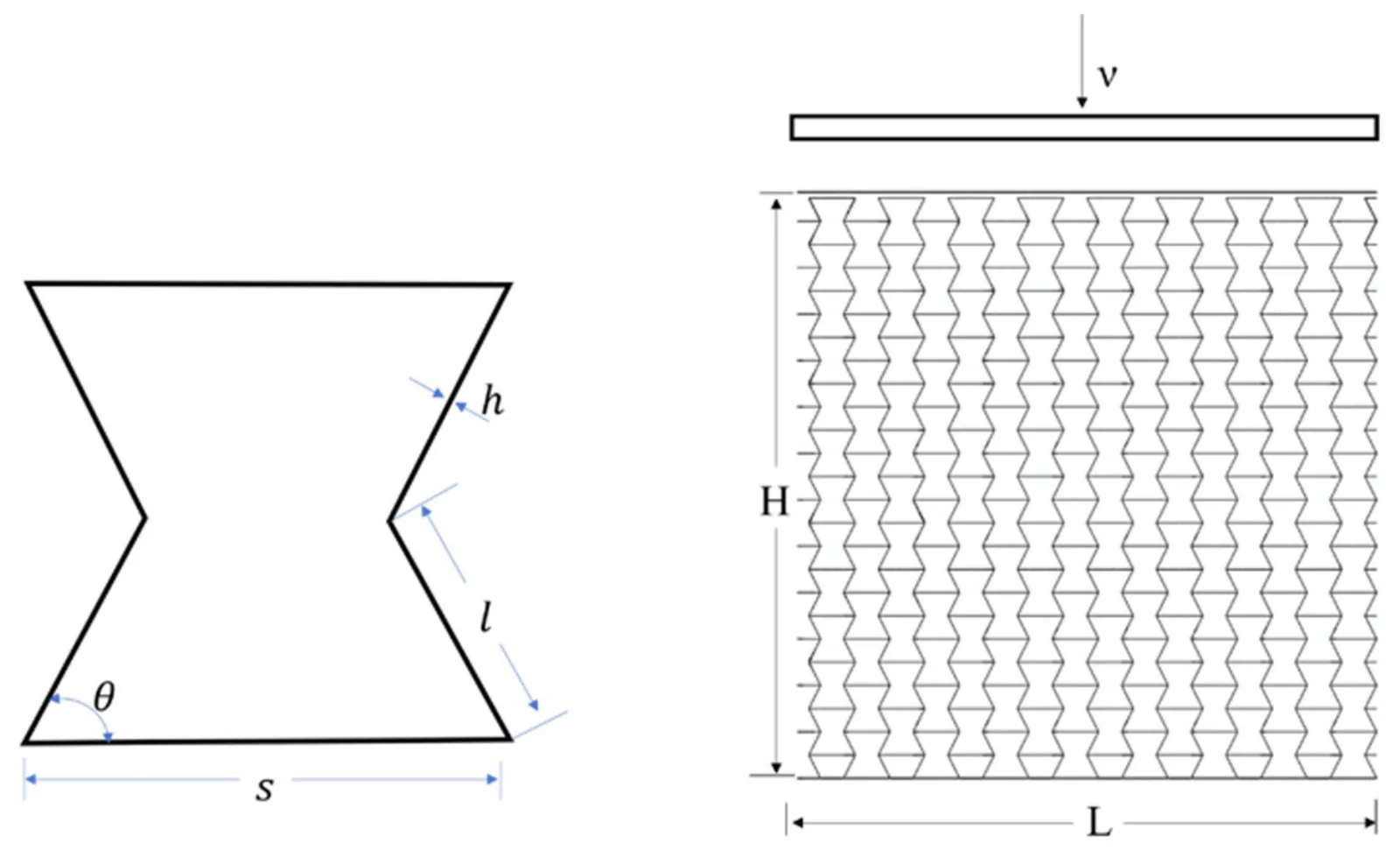
Geometric definition of the re-entrant honeycomb unit cell and the corresponding periodic cellular specimen under in-plane impact loading.

The numerical model was constructed using a two-dimensional periodic re-entrant honeycomb architecture. The unit-cell geometry was defined by the inclined ligament length *l*, horizontal base length *s*, cell-wall thickness *h*, and re-entrant angle *θ*. The geometric parameters were set to *l* = 4 mm, *s* = 8 mm, *h* = 1 mm, and *θ* = 60°, consistent with the re-entrant configuration previously employed by Tatlier [17] in a dynamic crushing study, as illustrated in Figure 1. Repetition of the unit cell generated a planar cellular specimen with overall in-plane dimensions of approximately 100 mm × 100 mm. An out-of-plane width of *W* = 15 mm was adopted throughout the model.

The finite element analyses were performed using CalculiX (Version 2.22, developed by Guido Dhondt and Klaus Wittig, Munich, Germany), an open-source finite element solver, with PrePoMax (Version 2.2.0, developed by Matej Borovinšek, Maribor, Slovenia) employed as the graphical interface for model preparation and postprocessing. The cellular walls were discretized using four-node quadrilateral shell elements with reduced integration (S4R). In CalculiX, these elements are internally expanded into eight-node hexahedral elements with reduced integration (C3D8R) during the solution procedure. A nominal element size of 2 mm was adopted, resulting in approximately 8605 shell elements and 9180 nodes. The cell-wall thickness was specified as *h* = 1 mm. This discretization was selected to capture large rotations, local ligament bending, plastic-hinge formation, and progressive collapse during dynamic in-plane compression. Particular attention was given to the re-entrant junctions, where substantial rotations and localized deformation are expected. The mesh was designed to represent these deformation mechanisms while maintaining computational efficiency during the explicit dynamic analyses.

**Figure 2:**
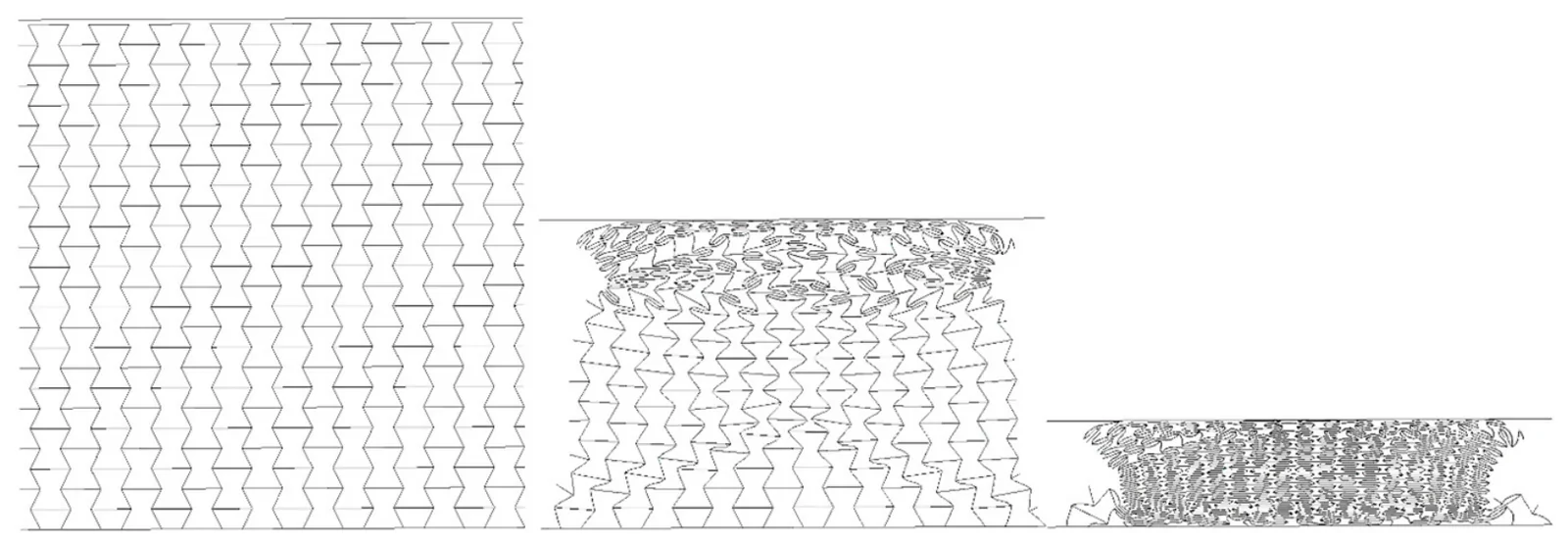
Representative successive deformation configurations of a re-entrant honeycomb during dynamic in-plane crushing, illustrating localized collapse and progressive compaction.

Dynamic crushing was simulated using an explicit dynamic formulation at impact velocities of 50, 75, and 100 m/s. A rigid striker was employed to compress the honeycomb along the in-plane loading direction. The nodes at the boundary opposite to the striker were fully constrained, while the impacted boundary was allowed to deform through contact with the rigid plate. Out-of-plane motion was restricted to preserve the intended two-dimensional crushing condition. Surface-to-surface contact and self-contact definitions were employed to account for interactions between the rigid striker and the cellular specimen, as well as progressive collapse and densification. A tangential friction coefficient of 0.3 was assigned to the contacting surfaces. The crushing response was evaluated over a striker displacement of 80 mm measured from the initial contact position as observed in Figure 2. The reaction force at the constrained boundary was extracted to determine the maximum crushing force, mean crushing force, and absorbed energy. The same numerical discretization, boundary conditions, and contact parameters were employed for the isotropic and functionally graded configurations to ensure a consistent comparison of their dynamic crushing responses.

**Figure 3:**
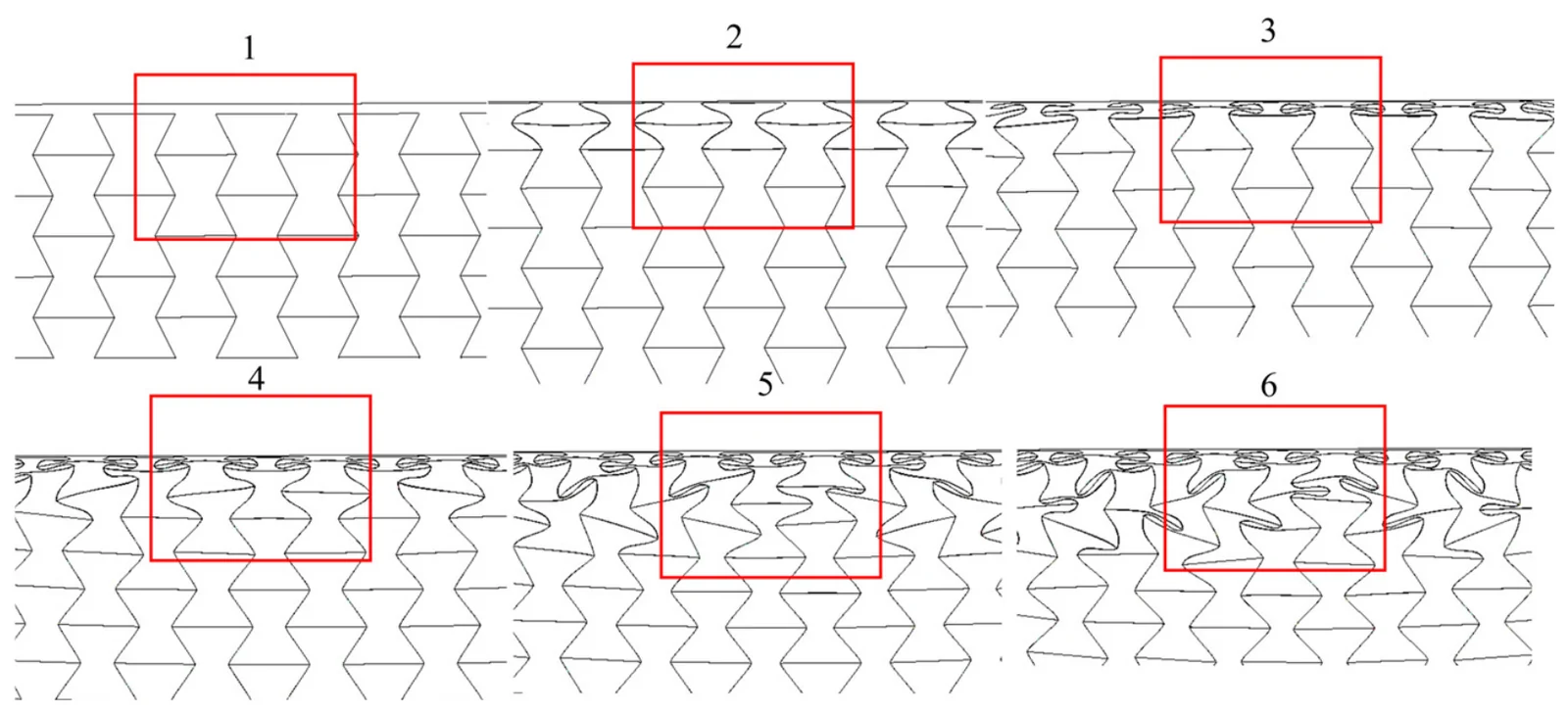
Representative deformation configurations of functionally graded (FGM) re-entrant honeycombs during dynamic in-plane crushing at an impact velocity of 100 m/s, illustrating the progression of localized deformation, cell-wall bending, plastic-hinge formation, and progressive compaction.

Representative deformation configurations of the functionally graded (FGM) re-entrant honeycomb subjected to dynamic in-plane crushing at an impact velocity of 100 m/s are presented in Figure 3. The six successive deformation stages illustrate the progression of cellular collapse, characterized by localized deformation, cell-wall bending, ligament rotation, and plastic-hinge formation. As compression proceeds, adjacent cell walls progressively approach one another, resulting in increased contact interactions and localized compaction. The collapse propagates non-uniformly through the cellular structure due to the combined effects of structural inertia, progressive plastic deformation, and the spatial variation in yield strength. These deformation mechanisms are consistent with the fluctuations observed in the corresponding force–displacement response. Since the numerical model employs a rate-independent elastic–perfectly plastic constitutive law without an explicit damage or fracture criterion, the observed local collapse mechanisms represent structural instability and plastic deformation rather than material fracture or element deletion.

The present investigation differs fundamentally from conventional geometrically graded re-entrant honeycombs. The cell dimensions, ligament thickness, and re-entrant angle were maintained constant throughout the entire cellular domain. Functional grading was introduced exclusively through the spatial variation in the constitutive properties of the parent material along the crushing direction. Consequently, the geometry and relative density of the cellular structure remain unchanged, and the differences observed between the isotropic and functionally graded configurations can be attributed directly to the imposed material-property distribution. This approach eliminates the simultaneous influence of geometric grading and provides a clearer assessment of the effect of constitutive gradation on deformation initiation, localization of collapse, crushing-force evolution, and energy-absorption behavior.

**Figure 4:**
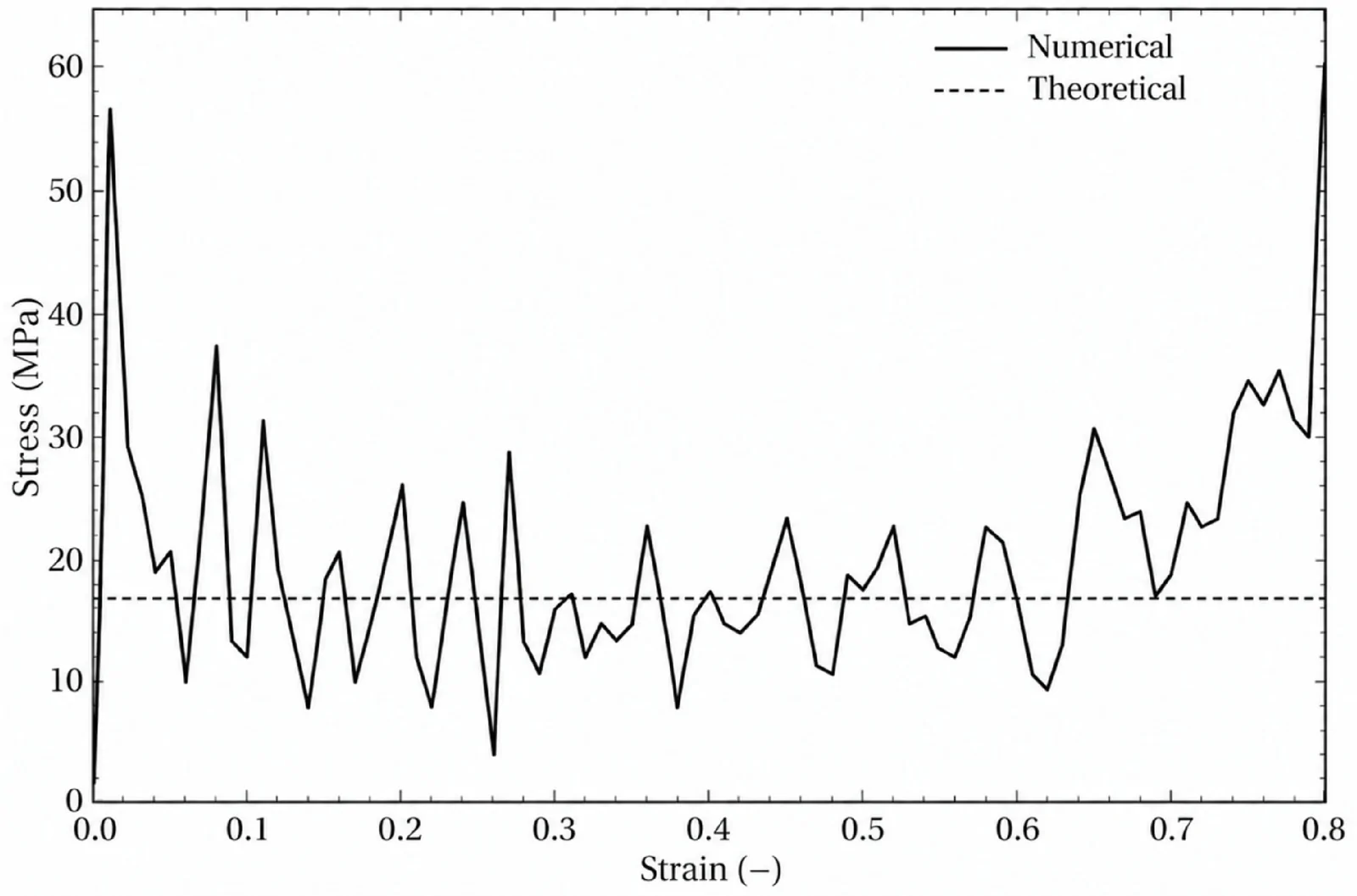
Numerical stress–strain response of the isotropic re-entrant honeycomb at an impact velocity of 100 m/s compared with the theoretical dynamic crushing stress predicted by Hu et al. [15].

The macroscopic nominal crushing stress and nominal compressive strain of the honeycomb structure were determined from the finite element results using the following relationships:

(1) $$\sigma_{\mathrm{nom}}=\frac{F_{R}}{A_{0}},\;A_{0}=W\times L$$

(2) $$\varepsilon_{\mathrm{nom}}=\frac{\delta }{H}$$

where *F_R_* is the magnitude of the reaction force obtained from the constrained boundary, *A*_0_ is the initial gross cross-sectional area perpendicular to the crushing direction, *L* is the specimen width, and *W* is the out-of-plane depth. Furthermore, *δ* represents the striker displacement measured from initial contact, while *H* denotes the initial specimen height along the crushing direction. For the present honeycomb structure, *L* = 100 mm, *W* = 15 mm, and *H* = 100 mm. These relationships were employed to convert the reaction force and striker displacement obtained from the finite element simulations into the corresponding nominal stress–strain response. Specifically, Equation (1) was used to calculate the nominal crushing stress, while Equation (2) was used to determine the nominal compressive strain. The resulting stress–strain response was subsequently used in the model validation presented in Section 2.3, where the numerical results were compared with the analytical dynamic crushing stress predicted by Hu et al. [15], as illustrated in Figure 4.

### 2.2. Material Model and Functional Grading

The constitutive behavior of the re-entrant cell walls was represented by a rate-independent elastic–perfectly plastic material model. The parent material was assigned a mass density of 2700 kg/m^3^, Young’s modulus of 70 GPa, Poisson’s ratio of 0.30, and a reference yield strength of 110 MPa. After yielding, the flow stress was assumed to remain constant and no strain hardening was introduced. This idealization was retained to isolate the effects of cellular deformation and material-strength grading from additional constitutive mechanisms. No strain-rate-dependent strengthening law was employed. Consequently, any variation in the crushing response with impact velocity arises from structural inertia, localized instability, progressive cell collapse, contact interactions, and densification rather than from intrinsic rate sensitivity of the parent material. The use of a rate-independent elastic–perfectly plastic material model was a deliberate modelling assumption intended to isolate the effects of spatial yield-strength grading on the dynamic crushing response. Although the structures were subjected to high impact velocities, the numerical formulation accounts for structural inertia, momentum transfer, localized plastic deformation, and progressive cell collapse. By excluding intrinsic strain-rate-dependent material strengthening, the effects of impact velocity and spatial variations in yield strength can be investigated independently of additional constitutive rate effects. Therefore, the observed velocity-dependent response is attributed to structural dynamic mechanisms rather than material strain-rate hardening. Nevertheless, the influence of intrinsic strain-rate sensitivity remains outside the scope of the present investigation.

Two constitutive configurations were considered while preserving the same re-entrant geometry. In the isotropic reference model, a uniform yield strength of 110 MPa was assigned throughout the entire cellular domain. In the functionally graded configuration, the elastic modulus, Poisson’s ratio, density, cell-wall thickness, out-of-plane width, and unit-cell dimensions were maintained identical to those of the isotropic model. Functional grading was introduced solely through a spatial variation in the local yield strength. Thus, the isotropic and FGM structures possess the same geometry, relative density, and total mass and differ only in the spatial distribution of plastic resistance.

The material gradient was prescribed along the global x-direction, which coincides with the crushing direction. A normalized spatial coordinate was defined as

(3) $$\xi =\frac{x-x_{\min}}{x_{\max}-x_{\min}},\;0\leq \xi \leq 1$$

where *x*_min_ and *x*_max_ denote the limits of the cellular specimen in the grading direction. The local yield strength was then defined by a linear relation,

*σ_y_*(*x*) = *σ_y_*_,*min*_ + (*σ_y_*_,*max*_ − *σ_y_*_,*min*_)*ξ* (4)

where *σ_y_*_,min_ = 55 MPa and *σ_y_*_,max_ = 165 MPa. Accordingly, the material strength increases progressively from the impact side toward the opposite end of the specimen, while the central region retains the reference value of 110 MPa. In normalized form, the corresponding strength factor varies from 0.50 to 1.50 relative to the isotropic material. The minimum and maximum yield strengths were deliberately selected symmetrically around the reference value of 110 MPa. Consequently, the prescribed linear distribution has a spatially averaged yield strength of 110 MPa along the grading direction, corresponding to the uniform yield strength of the ISO configuration. This selection was intended to establish a controlled numerical comparison in which the influence of spatially redistributing the plastic resistance could be investigated independently of changes in the reference strength, structural geometry, or total mass. The selected strength distribution therefore represents a prescribed numerical grading scheme rather than an experimentally calibrated material gradient.

The prescribed material distribution was implemented through field-dependent plasticity using the spatial variation defined by Equations (3) and (4). According to Equation (3), the position along the crushing direction is represented by the normalized coordinate *ξ*, ranging from 0 at the impact-side boundary to 1 at the opposite boundary. The corresponding local yield strength is determined from Equation (4), which defines a linear increase from 55 MPa to 165 MPa along the grading direction. For numerical implementation, reference field levels of 0.50, 0.75, 1.00, 1.25, and 1.50 were assigned to yield strengths of 55, 82.5, 110, 137.5, and 165 MPa, respectively. Intermediate values were obtained by interpolation through the spatial field, thereby reproducing the continuous yield-strength distribution prescribed by Equation (4), rather than a sequence of discrete material layers. Only the yield strength varies with position, while Young’s modulus, Poisson’s ratio, and density remain spatially uniform. Since each field state is defined by a single yield stress at zero plastic strain, the elastic–perfectly plastic behavior is preserved throughout the graded re-entrant honeycomb structure.

The present grading concept should be distinguished from geometric grading frequently employed in re-entrant honeycombs. Variations in re-entrant angle, ligament dimensions, wall thickness, or cell size also modify local relative density and structural stiffness and may alter the total mass. Such coupled changes make it difficult to identify the individual contribution of constituent-material strength to the global crushing response. In the present models, every re-entrant cell retains the same geometry and wall thickness. The grading field therefore changes the resistance to plastic deformation without changing the architecture through which the collapse front develops.

The gradient was oriented so that the lowest-strength region is located adjacent to the impacted boundary, whereas the yield resistance increases continuously in the crushing direction. The relatively compliant impact-side region promotes earlier yielding and provides a preferential zone for the initiation of plastic hinges and localized cell collapse. As the deformation progresses, the advancing collapse region encounters material with progressively higher yield resistance. The proposed strength distribution therefore redistributes crushing resistance along the deformation path and can influence the evolution of force transmission and progressive compaction without modifying the cellular topology.

Because the isotropic and FGM configurations have identical geometry, density, and mass, their responses can be compared without the additional influence of geometric or density grading. Differences in collapse localization, crushing-force evolution, energy absorption, peak-force development, and crush-force efficiency can therefore be attributed primarily to the imposed distribution of parent-material yield strength. This formulation provides a controlled basis for assessing material-based functional grading as a means of tailoring the dynamic crushing response of re-entrant auxetic honeycombs.

### 2.3. Model Validation

The numerical model was further assessed through the macroscopic stress–strain response obtained at an impact velocity of 100 m/s. The reaction force obtained from the finite element analysis was converted into nominal crushing stress using the effective loading area of the specimen, while the corresponding compressive strain was determined from the striker displacement relative to the initial specimen height. Considering the specimen dimensions adopted in the present analysis, the numerical force–displacement history was therefore expressed in stress–strain form to allow direct comparison with the analytical crushing-stress prediction. The resulting response is presented in Figure 4.

For comparison, the theoretical dynamic crushing stress was calculated using the high-velocity formulation developed by Hu et al. [15] for re-entrant hexagonal honeycombs. Their analytical treatment distinguishes between the plastic resistance associated with the collapse of the cellular walls and the additional contribution produced by cell-wall inertia under high-velocity loading. The model therefore predicts an increase in the effective crushing resistance as impact velocity increases, which is consistent with the localized layer-by-layer collapse observed in re-entrant cellular structures subjected to dynamic loading. For the present re-entrant geometry with *θ* = 60°, together with the corresponding geometric and material parameters, the analytical formulation gives a dynamic crushing stress of approximately 16.9 MPa at an impact velocity of 100 m/s. This value is represented by the horizontal dotted line in Figure 4. It should be noted that the analytical formulation proposed by Hu et al. [15] provides an approximate estimate of the characteristic dynamic crushing stress for a given honeycomb geometry, material properties, and impact velocity, rather than a strain-dependent stress–strain relationship. Therefore, the calculated theoretical value of approximately 16.9 MPa at an impact velocity of 100 m/s is represented by a horizontal reference line in Figure 4. This constant value should not be interpreted as indicating that the actual crushing stress remains unchanged throughout deformation. Instead, it serves as a theoretical benchmark against which the fluctuating numerical stress–strain response can be evaluated.

As observed in Figure 4, the numerical stress–strain response initially exhibits pronounced fluctuations associated with impact initiation and the onset of localized cell collapse. The repeated formation of plastic hinges, rotation of the inclined ligaments, and interaction between adjacent cell walls produce substantial oscillations in the instantaneous crushing stress. Such behavior is consistent with the dynamic deformation characteristics reported for re-entrant honeycombs under high-velocity loading. Hu et al. [15] showed that the deformation becomes increasingly localized near the impacted boundary as the crushing velocity increases and that the collapse subsequently propagates through the cellular layers.

The analytical crushing stress should therefore not be interpreted as an instantaneous stress value at every stage of deformation, but rather as a representative dynamic resistance associated with the characteristic cell-collapse process. Despite the pronounced numerical oscillations, the theoretical prediction lies within the sustained crushing-stress range over a substantial part of the progressive-collapse regime. At larger strains, the numerical stress increases as the structure approaches densification. This increase results from the progressive reduction in available void space, increased interaction between neighboring ligaments, and intensified self-contact during compaction. The numerical model consequently reproduces the principal stages expected during dynamic crushing of a re-entrant honeycomb: an initial transient response, an oscillatory progressive-collapse regime, and a final densification stage. The agreement between the theoretical dynamic crushing stress and the characteristic numerical stress level supports the consistency of the finite element model and its suitability for the subsequent comparison of isotropic and functionally graded re-entrant configurations.

## 3. Results and Discussion

The dynamic crushing behavior of the isotropic and functionally graded re-entrant honeycomb structures was investigated at impact velocities of 50, 75, and 100 m/s. The isotropic reference configuration, hereafter denoted as ISO, employed a spatially uniform parent-material yield strength of 110 MPa. In the functionally graded configuration, denoted as FGM, the yield strength increased continuously from 55 MPa at the impact side to 165 MPa toward the opposite boundary along the crushing direction. As described in Section 2.2, the cellular geometry, wall thickness, elastic properties, density, relative density, and total structural mass were unchanged between the two configurations. Therefore, differences in the numerical responses originate from the spatial distribution of plastic resistance rather than from variations in geometry or mass.

**Figure 5:**
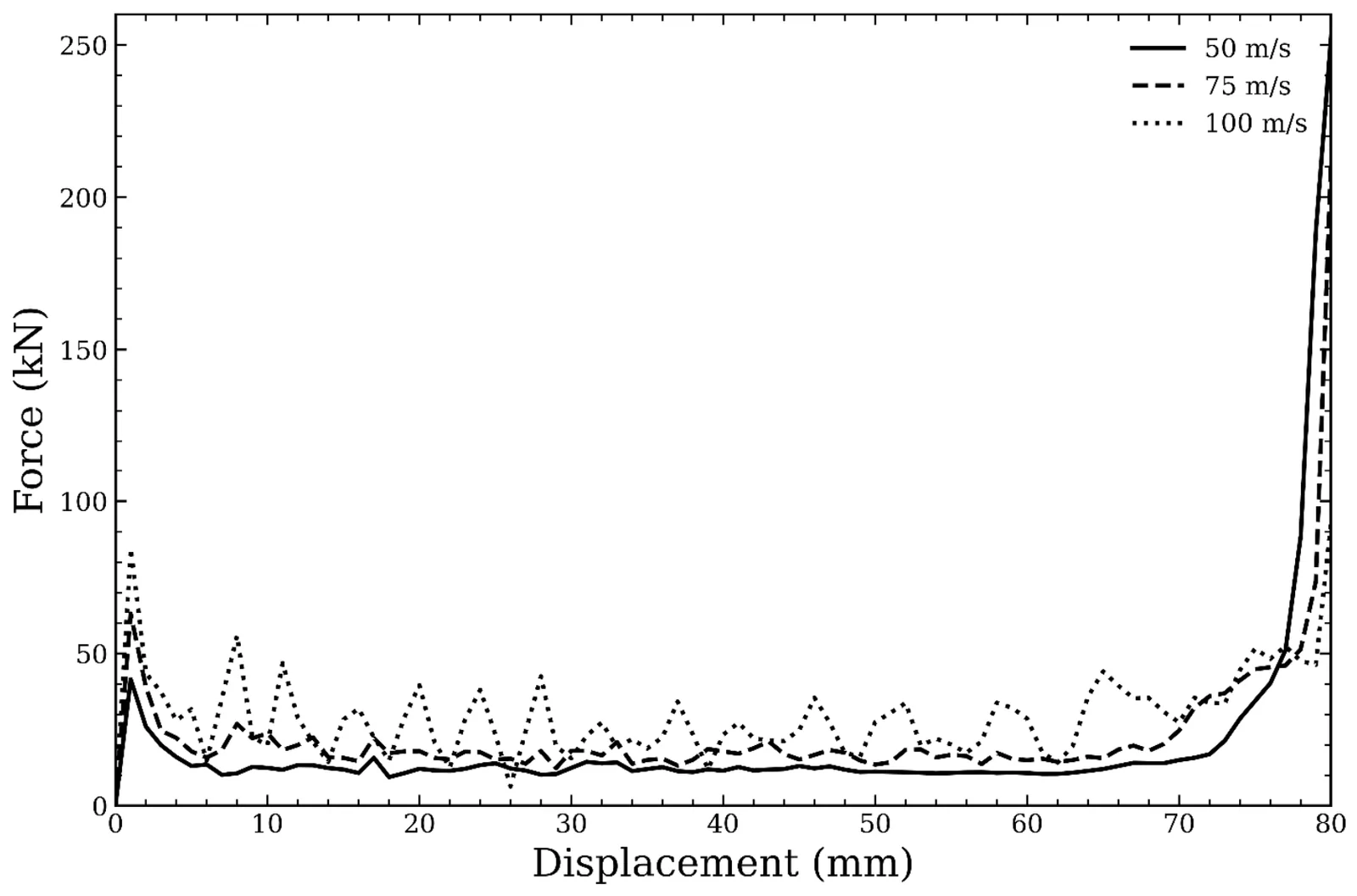
Force–displacement responses of the isotropic re-entrant honeycomb structure at impact velocities of 50, 75, and 100 m/s, illustrating the influence of impact velocity on progressive crushing and densification behavior.

Figure 5 presents the force–displacement responses of the isotropic re-entrant honeycomb at impact velocities of 50, 75, and 100 m/s. All three curves exhibit the characteristic sequence of an initial impact transient, an extended progressive-collapse stage, and a terminal increase in force associated with advanced cellular compaction. However, the relative contributions of these stages change substantially with impact velocity. At 50 m/s, the response is characterized by a comparatively low force level over most of the crushing displacement, followed by a pronounced increase close to the end of the prescribed displacement. The initial impact generates a local force peak, after which the force rapidly decreases and remains relatively low while successive re-entrant cells collapse.

Increasing the impact velocity to 75 m/s changes both the magnitude and the temporal distribution of the crushing force. The initial response becomes stronger, and the force level over the progressive-collapse region increases moderately. A terminal force rise remains evident; however, its magnitude is lower than that obtained at 50 m/s. At 100 m/s, the response becomes considerably more oscillatory. Numerous local force peaks develop over the entire crushing displacement, indicating repeated activation of localized deformation mechanisms within the re-entrant network. The sustained force level is also clearly higher than at the lower velocities, whereas the exceptionally large terminal peak observed at 50 m/s is no longer present.

The evolution observed in Figure 5 is reflected by the calculated mean and maximum crushing forces. The mean crushing force of the ISO structure increases from 18.83 kN at 50 m/s to 22.51 kN at 75 m/s and approximately 29.3 kN at 100 m/s. Thus, although the material model itself is rate-independent, the average resistance sustained by the re-entrant network increases markedly with impact velocity. From 50 to 100 m/s, the increase in mean crushing force is approximately 56%. Since no strain-rate strengthening is included in the constitutive model, this increase should primarily be associated with structural inertia, the changing localization of collapse, interactions between neighboring ligaments, and the different sequence in which the cellular layers participate in deformation.

An apparently opposite tendency is observed for the maximum crushing force. The ISO configuration develops *F*_max_ = 252.87 kN at 50 m/s and 211.78 kN at 75 m/s, whereas the maximum force at 100 m/s decreases to approximately 93 kN. This result demonstrates that the maximum force cannot be interpreted simply as a direct measure of impact severity. In the present re-entrant topology, the highest instantaneous force is strongly dependent on the stage at which extensive ligament interaction and near-locking of the cellular network occur. At the lower impact velocities, a substantial part of the network reaches an advanced compaction state near the end of the prescribed displacement, producing a sharp terminal force amplification. At higher velocity, deformation is more strongly localized and accompanied by repeated force oscillations. Consequently, greater average crushing resistance is sustained without the same degree of simultaneous global locking within the investigated displacement range.

The pronounced transient force peak observed at the onset of impact can be primarily interpreted in terms of the dynamic inertial resistance of the cellular structure. When the rigid striker contacts the honeycomb at high velocity, the cell walls adjacent to the impact boundary must undergo rapid acceleration and deformation within a very short time interval. According to the principle of linear momentum balance, a greater rate of momentum transfer requires a larger instantaneous force. Consequently, the resistance measured during the initial impact is not governed solely by the plastic collapse strength of the cell walls but also includes the additional force required to accelerate the participating structural mass.

At an impact velocity of 100 m/s, the short loading time restricts the ability of the cellular network to deform and redistribute the applied load throughout the structure. The deformation consequently tends to concentrate near the impacted boundary, where the rapid acceleration of the inclined cell walls and the development of localized plastic hinges generate a pronounced initial force peak. This behavior can be interpreted as a form of dynamic inertial constraint, often associated with inertial locking in cellular structures. Unlike conventional geometric locking caused by extensive cell-wall contact during densification, this early-stage resistance originates primarily from the inertia of the rapidly deforming cell walls and the limited time available for progressive collapse.

The initial force peak may also be influenced by the propagation and reflection of transient stress waves generated at the moment of impact. Furthermore, the transition from the initial elastic response to localized yielding and plastic-hinge formation contributes to the subsequent reduction in crushing force. These mechanisms are not mutually exclusive. Nevertheless, the strong dependence of the initial response on impact velocity, together with the use of a rate-independent elastic–perfectly plastic material model, supports the interpretation that structural inertia and localized dynamic deformation play important roles in the development of the initial peak.

The force peaks observed during impact initiation and terminal densification arise from different physical mechanisms. The initial force peak is primarily associated with structural inertia, whereas the terminal force increase is governed by cell-wall accumulation, contact interactions, and geometric densification.

At 100 m/s, inertial effects become considerably more pronounced due to the rapid acceleration of the cellular structure and the higher rate of momentum transfer during impact initiation. These stronger inertial effects generate a pronounced initial force peak. As compression progresses, collapsed cell walls accumulate and increasingly come into contact with one another, producing an additional rise in force near the end of the crushing process due to geometric densification.

Therefore, the initial and terminal force peaks are governed by different mechanisms. The initial dynamic response becomes increasingly influenced by structural inertia and rapid momentum transfer as impact velocity increases, whereas the force rise during terminal compression results from the accumulation, contact, and densification of collapsed cell walls. Since the parent material is modeled as rate-independent elastic–perfectly plastic, the stronger initial inertial response at higher impact velocity is attributed to structural dynamics rather than intrinsic material strain-rate hardening.

**Figure 6:**
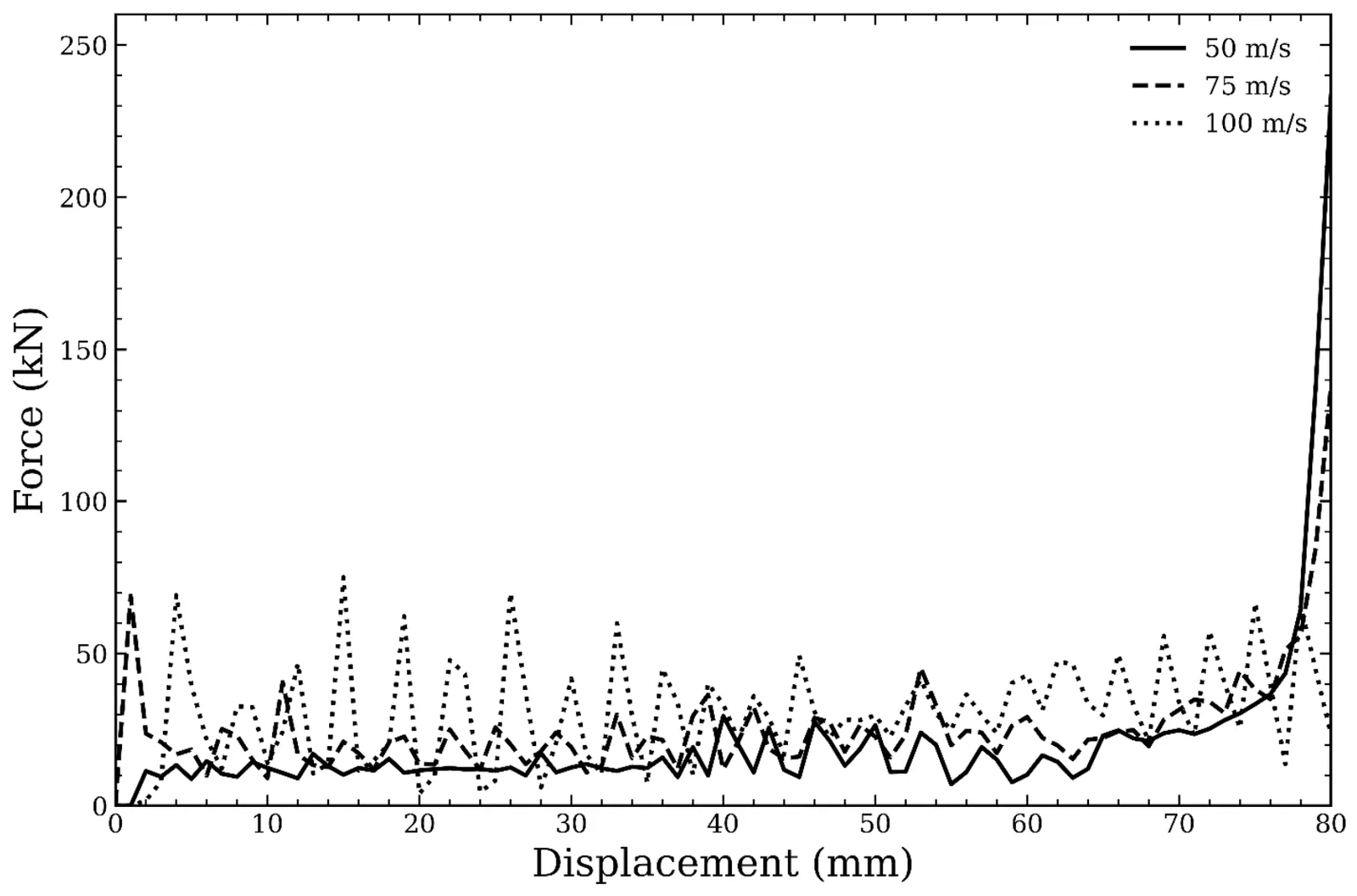
Force–displacement responses of the functionally graded re-entrant honeycomb structure at impact velocities of 50, 75, and 100 m/s, showing the effect of impact velocity on the evolution of crushing resistance.

Figure 6 shows the corresponding force–displacement responses of the functionally graded re-entrant structures. The general influence of impact velocity is similar to that observed for the isotropic cases, but the individual force histories reveal important differences associated with the prescribed strength gradient. At 50 m/s, the FGM structure initially deforms at relatively low force levels. Local oscillations develop during progressive crushing, followed by a pronounced force rise as the structure approaches the end of the compression stroke. At 75 m/s, the intermediate crushing regime shifts toward higher forces, while the terminal peak becomes substantially lower. The 100 m/s response displays the largest number and magnitude of local oscillations over the progressive-collapse region, indicating more frequent activation of individual cellular collapse events.

The increasing level of oscillation with impact velocity is especially relevant to the graded configuration because different regions of the structure possess different yield resistances. Collapse initiates in the relatively weak impact-side material and subsequently advances toward progressively stronger regions. As the crushing front encounters increasing local yield strength, the force required to activate subsequent cell-wall mechanisms changes continuously. This provides a reasonable mechanical explanation for the repeated peaks and valleys observed in the force histories. The local force response is therefore governed jointly by the re-entrant kinematics and by the position-dependent plastic resistance of the cell walls.

The FGM mean crushing force increases from 19.35 kN at 50 m/s to 25.10 kN at 75 m/s and approximately 30.9 kN at 100 m/s. The corresponding increase between the lowest and highest investigated velocities is close to 60%. This velocity sensitivity is comparable to, and slightly greater than, that obtained for the isotropic configuration. The increasing mean force confirms that the effect of inertia and deformation localization remains important even though the yield-strength distribution itself is independent of impact velocity.

In contrast, the maximum force of the graded structure decreases substantially with increasing velocity. *F*_max_ decreases from 233.44 kN at 50 m/s to 138.18 kN at 75 m/s and to approximately 75 kN at 100 m/s. Thus, the high-velocity configuration sustains greater average resistance but experiences a much lower extreme force than the 50 m/s case. This combination again indicates that velocity changes the deformation sequence rather than merely scaling the magnitude of the entire force history. In particular, the pronounced terminal densification event occurring at the lower velocity becomes progressively less dominant relative to the distributed crushing resistance as velocity increases.

**Figure 7:**
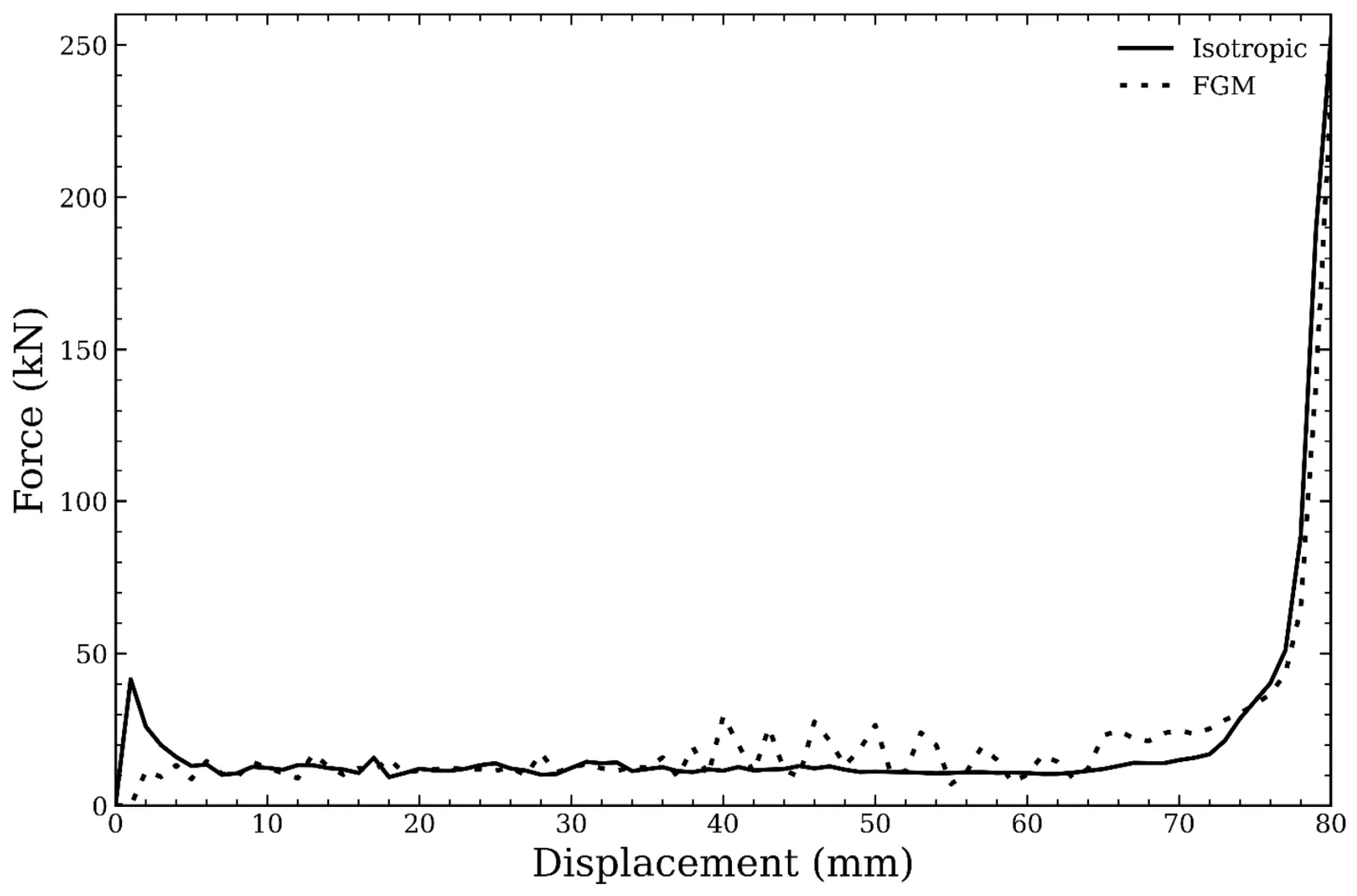
Comparison of the force–displacement responses of isotropic and functionally graded re-entrant honeycomb structures at an impact velocity of 50 m/s.

A direct comparison of the ISO and FGM configurations at 50 m/s is presented in Figure 7. At this velocity, the overall responses of the two configurations are relatively close. Both structures exhibit an initial transient followed by an extended low-force progressive-collapse regime and a sharp terminal increase as the cellular network becomes highly compacted. Nevertheless, closer examination shows that the FGM response contains more pronounced intermediate oscillations and generally maintains slightly greater force over portions of the progressive-collapse region.

At 50 m/s, the isotropic structure develops a maximum crushing force of 252.87 kN, whereas the FGM configuration reaches 233.44 kN. Functional grading therefore reduces the maximum force by approximately 7.68%. Although this reduction is relatively modest compared with those obtained at higher velocities, it occurs without a corresponding reduction in sustained crushing resistance. The mean crushing force actually increases from 18.83 kN for the ISO structure to 19.35 kN for the FGM configuration, corresponding to an increase of approximately 2.77%.

This difference is also reflected in the energy absorbed during crushing. The ISO structure absorbs 1506.42 J, whereas the FGM configuration absorbs 1548.02 J. Thus, functional grading produces an increase of approximately 2.76% in absorbed energy at 50 m/s. The combination of slightly greater mean force and lower maximum force results in an improvement in crush force efficiency. The CFE increases from 0.0745 for the isotropic structure to 0.0829 for the graded model, representing an improvement of approximately 11.3%.

These results are important because the effect of grading in the re-entrant geometry is not equivalent to simply reducing the overall material strength. If the entire cellular structure had been uniformly weakened, both the mean and maximum forces would be expected to decrease. Instead, the prescribed distribution combines a low-strength region near the impact side with progressively stronger material deeper within the network. The weaker region assists the initiation of deformation, while the stronger regions encountered later maintain or increase the sustained resistance. Consequently, the FGM structure at 50 m/s is able to absorb slightly more energy while generating a somewhat smaller peak load.

**Figure 8:**
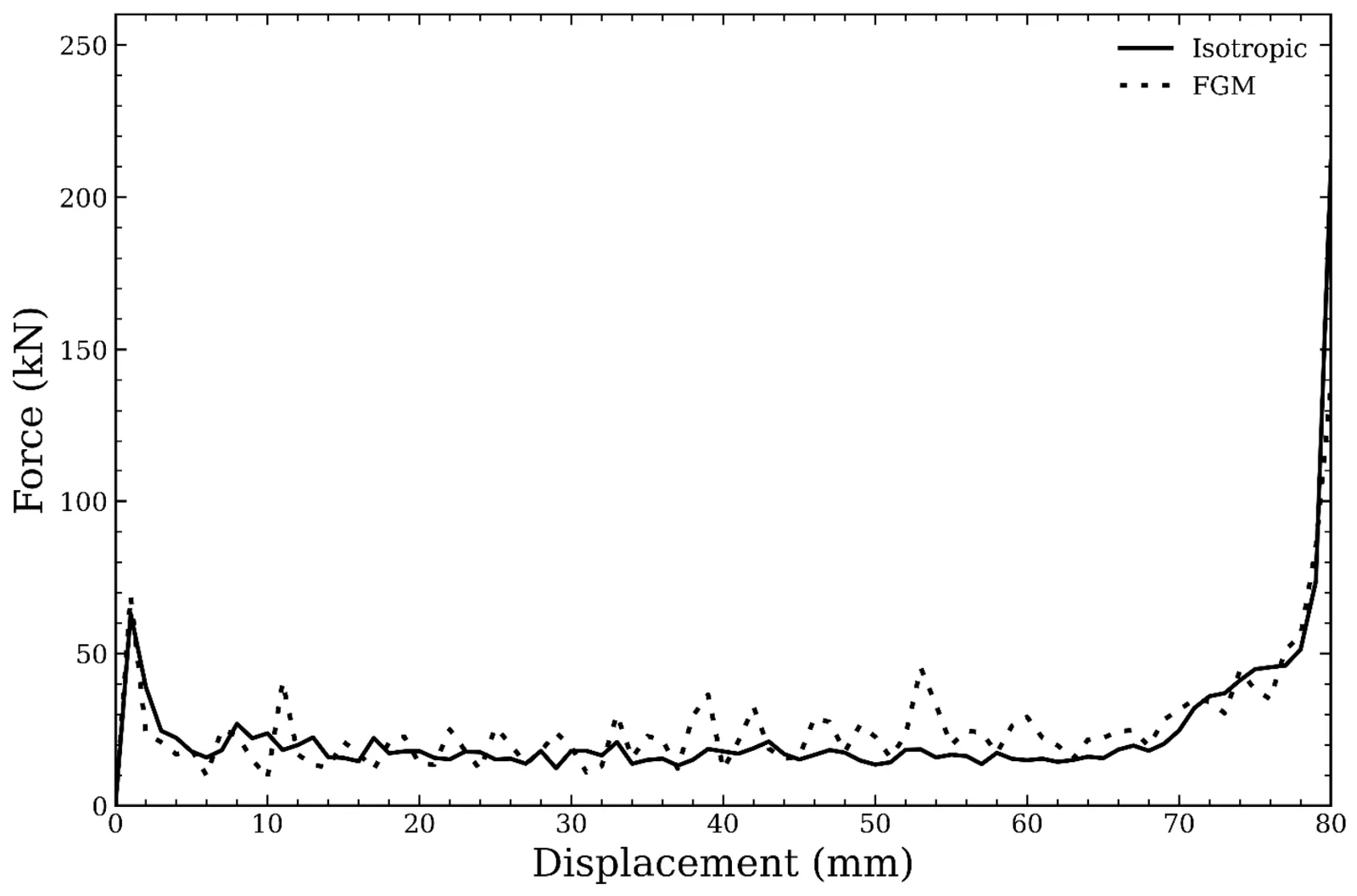
Comparison of the force–displacement responses of isotropic and functionally graded re-entrant honeycomb structures at an impact velocity of 75 m/s.

Figure 8 compares the two configurations at an impact velocity of 75 m/s. The distinction between ISO and FGM becomes considerably more pronounced at this velocity. The isotropic curve generally remains at lower force levels through parts of the central crushing interval and then undergoes a strong force increase as the displacement approaches 80 mm. In contrast, the FGM structure develops larger and more frequent local force oscillations throughout the progressive-collapse region but experiences a substantially smaller terminal force amplification.

The maximum force decreases from 211.78 kN for the isotropic structure to 138.18 kN for the FGM configuration. This corresponds to a reduction of approximately 34.75%, substantially greater than the reduction obtained at 50 m/s. At the same time, the mean crushing force increases from 22.51 to 25.10 kN, representing an enhancement of approximately 11.51%. The graded structure therefore develops a lower extreme force while sustaining a greater average resistance over the prescribed crushing displacement.

The absorbed energy follows the same trend as the mean crushing force. The ISO model absorbs 1800.52 J, whereas the FGM structure absorbs 2007.71 J, providing an increase of approximately 11.51%. This improvement is considerably larger than the 2.76% increase obtained at 50 m/s. The results indicate that the benefit of spatially varying material strength becomes more pronounced when the deformation is sufficiently dynamic for inertia and localized crushing to interact strongly with the prescribed material gradient.

The CFE values provide an especially clear representation of this change. At 75 m/s, the CFE increases from 0.1063 for the isotropic structure to 0.1816 for the graded configuration. This corresponds to an improvement of approximately 70.8%. The unusually large CFE enhancement results from the simultaneous increase in mean force and reduction in maximum force. In other words, a greater fraction of the maximum load-carrying capacity of the graded structure is converted into useful sustained crushing resistance rather than being concentrated in a short-duration force peak.

This behavior is particularly desirable from a crashworthiness perspective. High energy absorption alone does not necessarily indicate an efficient impact absorber if the same structure generates an excessive maximum transmitted force. At 75 m/s, the graded re-entrant configuration improves both sides of this balance: more energy is dissipated over the crushing stroke while the maximum instantaneous force is considerably reduced. The strength gradient therefore changes not only the magnitude of crushing resistance but also its distribution over displacement.

**Figure 9:**
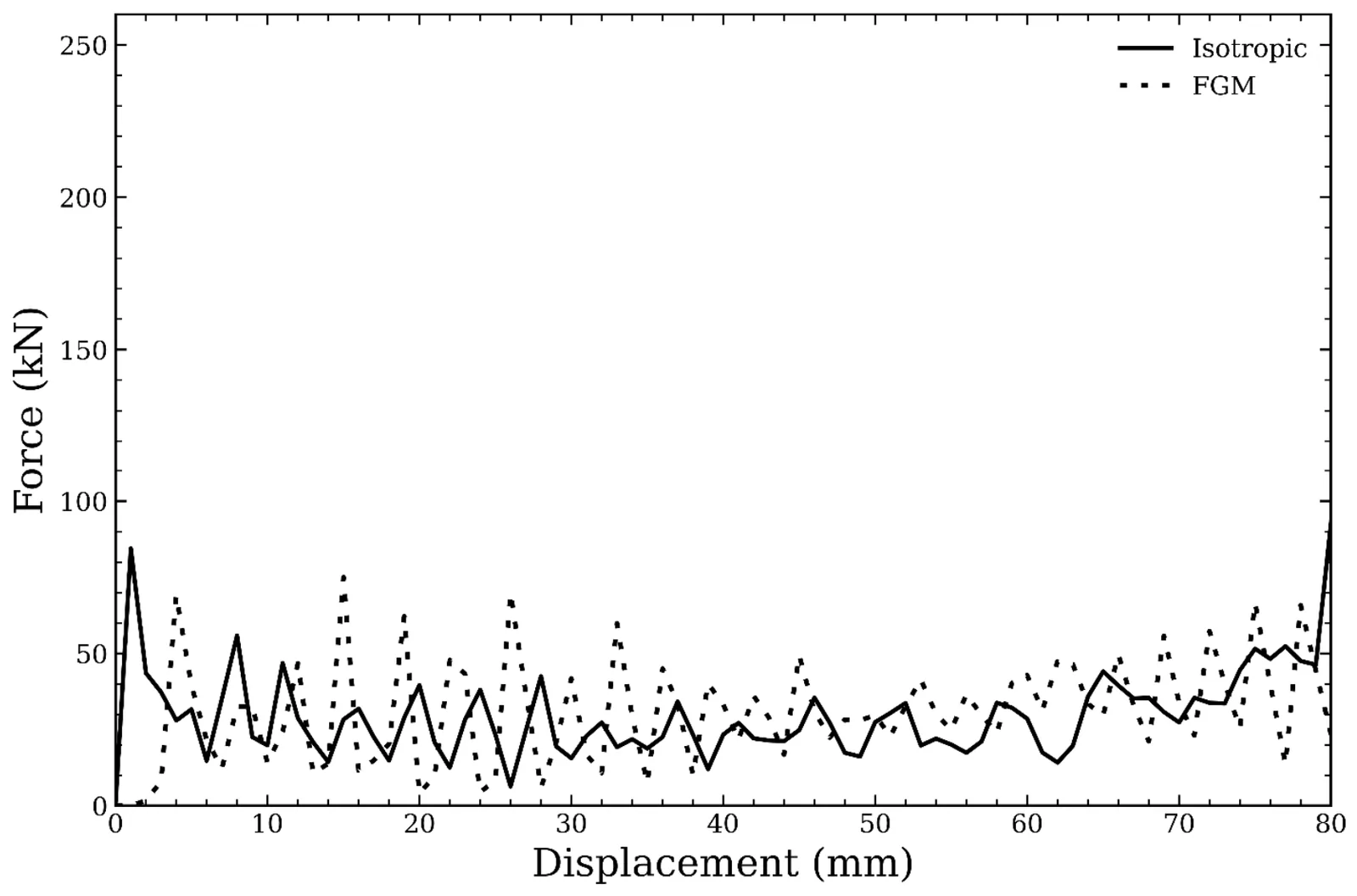
Comparison of the force–displacement responses of isotropic and functionally graded re-entrant honeycomb structures at an impact velocity of 100 m/s.

The ISO and FGM responses at 100 m/s are compared in Figure 9. At the highest investigated velocity, the overall behavior differs substantially from that observed at 50 m/s. Instead of a long low-force regime followed by an overwhelmingly dominant final densification peak, both configurations exhibit strong force oscillations throughout the crushing process. The isotropic curve undergoes repeated local peaks and valleys, whereas the FGM curve generally operates at a higher force level over much of the intermediate displacement range.

The FGM configuration frequently exceeds the isotropic force during progressive collapse, particularly over the middle and later portions of the crushing stroke. This explains the higher mean force and absorbed energy of the graded model. The mean crushing force is approximately 29.3 kN for the ISO configuration and 30.9 kN for the FGM structure. Functional grading therefore provides an increase of roughly 5–6% in sustained crushing resistance at this velocity. The absorbed energies are approximately 2.35 kJ and 2.48 kJ for the ISO and FGM structures, respectively, indicating a similar improvement in energy dissipation.

At the same time, the largest instantaneous force remains lower in the functionally graded model. The maximum force decreases from approximately 93 kN for the ISO configuration to approximately 75 kN for the FGM structure, corresponding to a reduction of about 19%. The magnitude of this reduction is smaller than the 34.75% obtained at 75 m/s but remains substantially greater than the difference observed at 50 m/s. The CFE increases from approximately 0.315 to 0.412, giving an improvement of approximately 31%.

The force histories at 100 m/s illustrate particularly well the mechanism by which material grading influences the re-entrant topology. The FGM configuration does not simply shift the complete force curve downward. Instead, it redistributes resistance. Local force levels are often higher throughout progressive crushing because increasingly strong material is activated as deformation advances, while the final extreme force remains below that of the uniform structure. This redistribution explains why the graded model simultaneously achieves greater absorbed energy and higher CFE despite having a lower maximum load.

**Figure 10:**
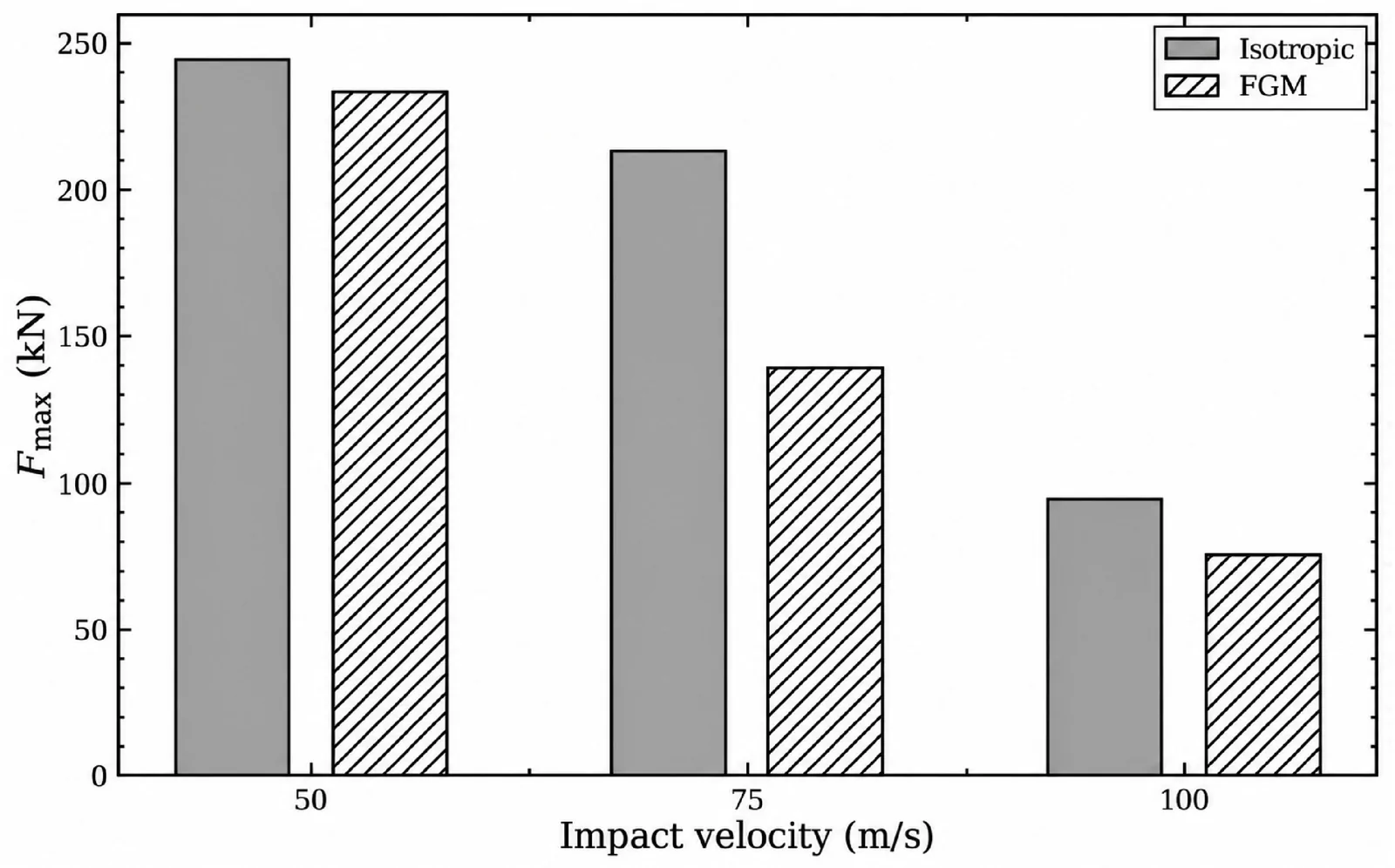
Comparison of maximum crushing force, *F_max_*, of isotropic and functionally graded re-entrant honeycomb structures at different impact velocities.

The velocity dependence of the maximum crushing force is summarized in Figure 10. A strong decrease in *F*_max_ is observed with increasing impact velocity for both material configurations. The isotropic structure decreases from 252.87 kN at 50 m/s to 211.78 kN at 75 m/s and approximately 93 kN at 100 m/s. The FGM structure follows the same overall trend, decreasing from 233.44 to 138.18 kN and finally to approximately 75 kN.

Between 50 and 100 m/s, the maximum force decreases by approximately 63% for the isotropic configuration and approximately 68% for the FGM structure. This trend might initially appear counterintuitive because impact severity increases with velocity. However, *F*_max_ in a cellular structure is not controlled solely by the instantaneous kinetic loading. In the present models, the largest force at lower velocity occurs during the severe terminal compaction of the network. As velocity increases, localized dynamic crushing and cell-wall inertia alter the sequence in which cells collapse and interact. Consequently, the structure sustains higher force throughout a larger fraction of the displacement while avoiding the same magnitude of collective terminal locking within the prescribed crushing stroke.

Functional grading reduces *F*_max_ at all investigated velocities. The reduction is approximately 7.68% at 50 m/s, increases substantially to 34.75% at 75 m/s, and remains about 19% at 100 m/s. Hence, the magnitude of the grading benefit is velocity-dependent and reaches its largest value at the intermediate investigated velocity. The non-monotonic relative reduction indicates that interaction between the material-strength gradient and the evolving collapse mechanism cannot be represented by a simple scaling relation. The stage at which stronger regions become involved, the degree of contact accumulation, and the location of the active crushing front all contribute to the maximum load.

**Figure 11:**
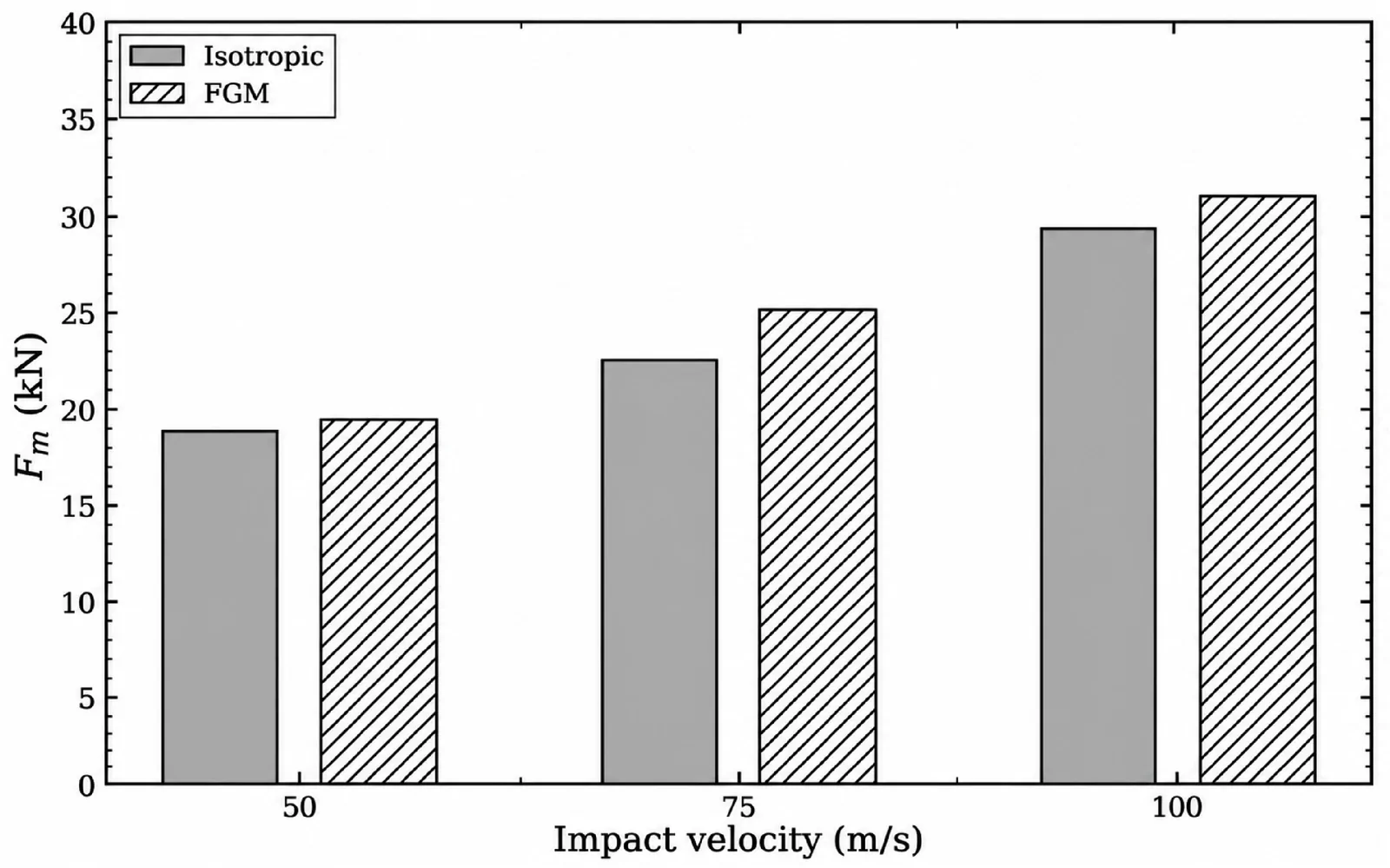
Comparison of mean crushing force, *F_m_*, of isotropic and functionally graded re-entrant honeycomb structures at different impact velocities.

Figure 11 presents the variation in mean crushing force, *F_m_*, with impact velocity. In contrast to the declining maximum force, *F_m_* increases consistently with velocity for both configurations. The isotropic values increase from 18.83 to 22.51 kN and then to approximately 29.3 kN. The FGM values increase from 19.35 to 25.10 kN and approximately 30.9 kN. Thus, higher velocity produces a clear enhancement of the sustained resistance of the re-entrant cellular structure.

The difference between *F_m_* and *F*_max_ is central to understanding the present results. While the maximum force is dominated by isolated events such as severe compaction or extensive simultaneous contact, the mean force reflects the resistance maintained over the complete crushing displacement. Increasing velocity raises the latter through inertial effects and more intense dynamic interaction between cell walls even though the final peak becomes smaller. From 50 to 100 m/s, *F_m_* increases by approximately 56% for the ISO structure and approximately 60% for the FGM structure.

The FGM configuration exhibits a higher mean force than the isotropic model at every velocity. The increase is approximately 2.76% at 50 m/s, 11.51% at 75 m/s, and about 5–6% at 100 m/s. The greatest relative improvement again occurs at 75 m/s. This result suggests that the interaction between collapse-front propagation and the increasing local yield resistance is particularly effective at this velocity. The graded structure initially benefits from the compliant impact-side region, while progressively stronger material provides greater resistance during subsequent crushing.

**Figure 12:**
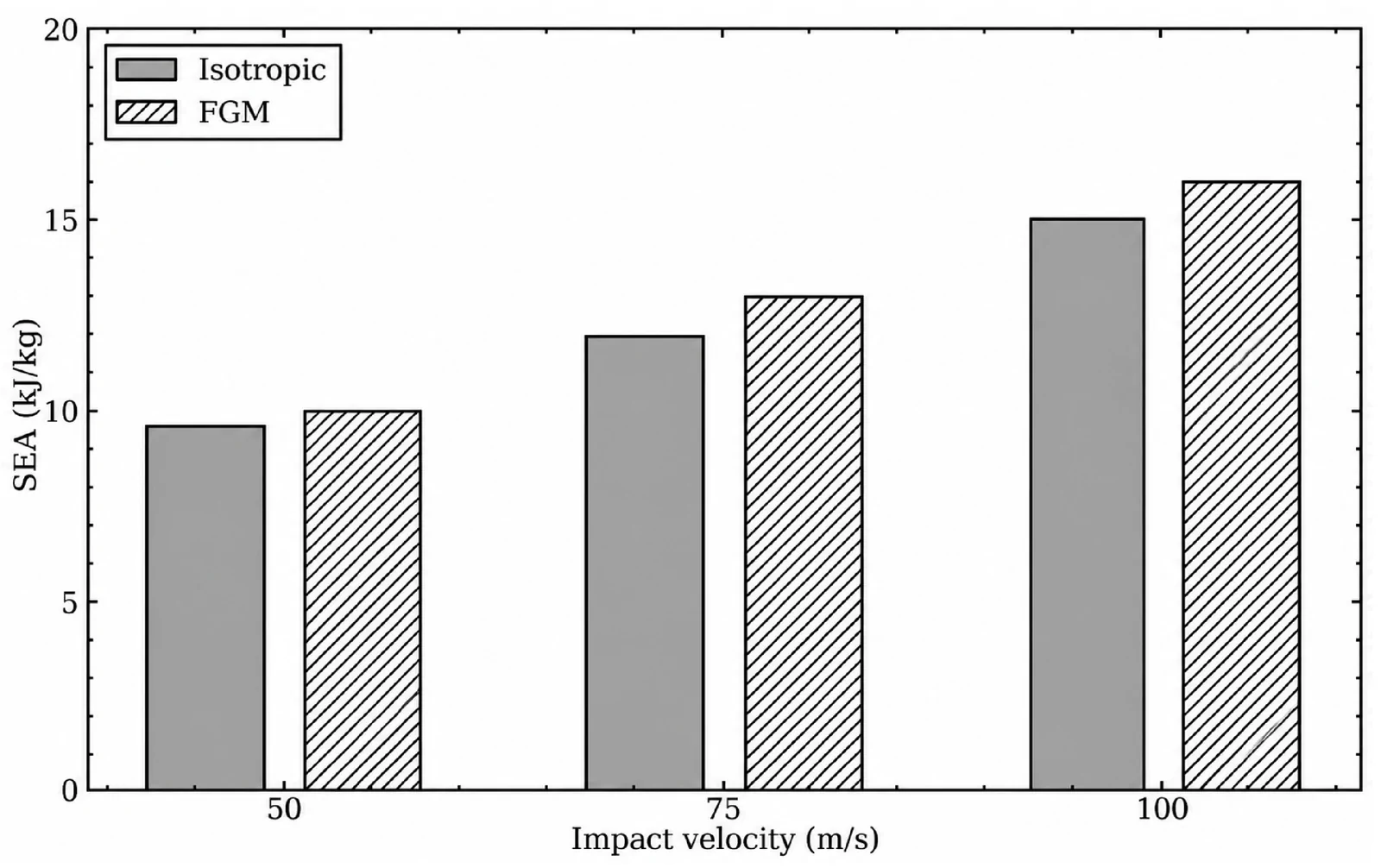
Comparison of specific energy absorption, SEA, of isotropic and functionally graded re-entrant honeycomb structures at different impact velocities.

The specific energy absorption (SEA) results shown in Figure 12 follow the same overall trend as the mean crushing force, consistent with the identical structural mass of the two configurations.

The energy absorbed by the isotropic structure increases from 1506.42 J at 50 m/s to 1800.52 J at 75 m/s and approximately 2345 J at 100 m/s. The corresponding FGM values are 1548.02 J, 2007.71 J, and approximately 2475 J. Because the displacement interval used for evaluating the two configurations is identical, the close relationship between absorbed energy and mean crushing force is expected.

Unlike the behavior previously associated with many graded structures in which peak-force reduction is achieved at the expense of energy absorption, the present re-entrant FGM configuration exhibits higher absorbed energy at all three investigated velocities. At 50 m/s, the increase is relatively small at approximately 2.76%. At 75 m/s, the improvement reaches approximately 11.51%, while at 100 m/s the increase remains approximately 5–6%. Thus, material-strength grading does not create a direct trade-off between energy absorption and force mitigation in the present topology. Instead, a modest-to-moderate improvement in energy absorption is obtained simultaneously with a reduction in maximum crushing force.

This distinction is particularly important when evaluating the effectiveness of the proposed grading strategy. The low-strength region adjacent to the impact side permits deformation to initiate without generating an excessive early resistance. Once collapse progresses into regions possessing greater yield strength, the force required to continue plastic deformation increases. As a result, resistance is distributed more effectively along the displacement history. Energy dissipation is enhanced because stronger material remains available to participate in later stages of crushing, while the initial compliant region helps prevent the development of an excessively high localized force.

The influence of velocity on specific energy absorption is also substantial. From 50 to 100 m/s, SEA increases by approximately 56% for the ISO configuration and nearly 60% for the FGM configuration. Since the material model is rate-independent, the increase cannot be attributed to strain-rate hardening. It instead reflects changes in the structural deformation process. At higher velocities, greater inertial resistance, more frequent local collapse events, and stronger interaction among the cellular ligaments lead to greater work being dissipated over the prescribed displacement.

**Figure 13:**
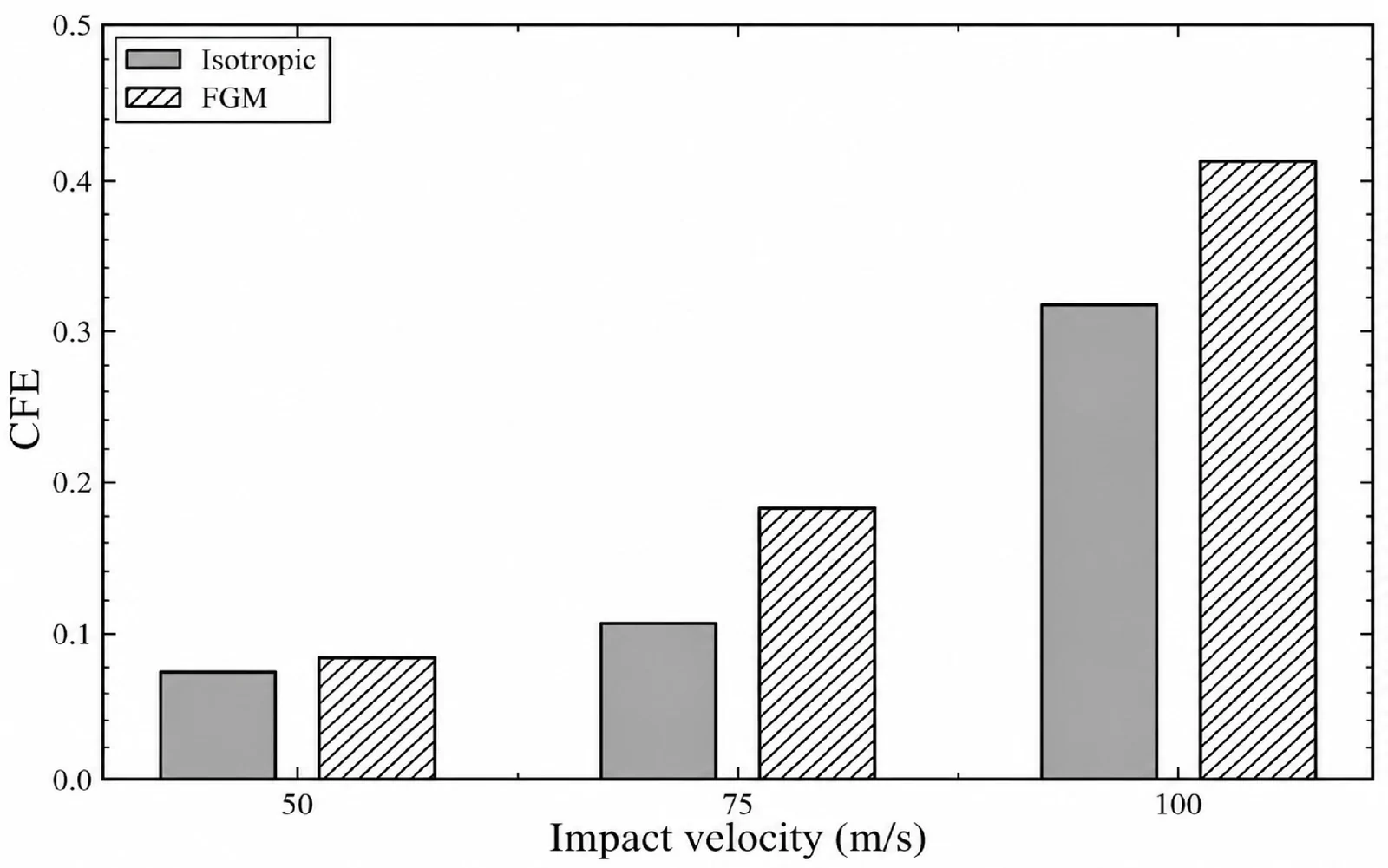
Comparison of crush force efficiency, CFE, of isotropic and functionally graded re-entrant honeycomb structures at different impact velocities.

Figure 13 compares the crush force efficiency of the two configurations. CFE displays the strongest velocity dependence among the evaluated crashworthiness parameters. For the isotropic model, CFE increases from 0.0745 at 50 m/s to 0.1063 at 75 m/s and to approximately 0.315 at 100 m/s. The corresponding values for the FGM structure are 0.0829, 0.1816, and approximately 0.412.

The rapid increase in CFE with impact velocity results directly from the opposite trends of *F_m_* and *F*_max_. The mean crushing force increases with velocity, whereas the maximum force decreases. Consequently, the ratio between useful sustained resistance and the largest transmitted load becomes progressively more favorable. Between 50 and 100 m/s, the isotropic CFE increases by more than four times its initial value, while the FGM CFE increases by almost five times.

Functional grading improves CFE at every investigated impact velocity. The increase relative to the ISO structure is approximately 11.3% at 50 m/s, reaches approximately 70.8% at 75 m/s, and remains approximately 31% at 100 m/s. The exceptionally large improvement at 75 m/s arises from the combined 11.5% increase in mean force and 34.8% reduction in maximum force. At 100 m/s, both structures already possess substantially improved CFE because the severe low-velocity densification peak is strongly reduced; nevertheless, the graded configuration maintains an additional advantage.

Considering Figure 10, Figure 11, Figure 12 and Figure 13 together reveals an important characteristic of the re-entrant FGM concept. Functional grading consistently shifts the crushing response toward a more favorable combination of lower extreme load and greater sustained resistance. The maximum crushing force is reduced, whereas the mean force and absorbed energy increase. This is fundamentally different from an indiscriminate reduction in material strength, which would be expected to decrease both maximum and average resistance. The present gradient instead redistributes strength spatially so that different sections of the cellular network perform different functions during crushing.

The low-yield-strength region near the impact side provides a relatively compliant initiation zone. Plastic rotation of the re-entrant ligaments can begin in this region at comparatively low resistance, reducing the tendency for a severe force peak to develop during the initial stages of deformation. With continued crushing, the active deformation region advances toward material of higher yield strength. These stronger regions resist subsequent ligament rotation and collapse more effectively, thereby maintaining or increasing the average force and energy dissipated during the remaining crushing process.

This mechanism also helps explain the strong dependence of the grading benefit on impact velocity. At 50 m/s, deformation proceeds sufficiently gradually that the distinction between the uniform and graded strength distributions remains comparatively small over much of the crushing stroke. Consequently, only modest changes in the global metrics are obtained. At 75 m/s, interaction between the propagating collapse process and the spatial strength distribution becomes considerably stronger. The graded structure can sustain a greater mean resistance while avoiding the large maximum force associated with the isotropic configuration. At 100 m/s, inertia becomes increasingly dominant and both structures exhibit strongly localized and oscillatory collapse. Although grading remains beneficial, the relative difference between the two models is smaller than at 75 m/s for some performance indicators.

The decrease in *F*_max_ with velocity should therefore be considered together with the changing shape of the force–displacement curves rather than interpreted in isolation. The low-velocity structures develop very high forces only near the end of the crushing displacement, when extensive material accumulation and contact occur. These short-duration force peaks strongly influence *F*_max_ but contribute comparatively little to the area under the complete force–displacement curve. Conversely, the higher-velocity responses develop elevated force over a much larger portion of the displacement history. As a result, SEA and *F_m_* increase even though the highest individual peak becomes smaller.

The same observation clarifies why CFE increases so dramatically with velocity. A structure that sustains a relatively modest force through most of its collapse but develops a very large terminal densification load necessarily exhibits poor crush force efficiency. This is the behavior observed most clearly at 50 m/s. At higher velocities, the difference between sustained crushing force and the maximum force becomes substantially smaller. The resulting response is more efficient in the sense that less of the structure’s peak-load-carrying capacity is concentrated in an isolated extreme event.

From a design perspective, the FGM re-entrant configuration is therefore advantageous not because it maximizes one isolated crashworthiness parameter but because it improves the balance among several competing quantities. At all velocities, its maximum force is lower than that of the isotropic configuration. At the same time, its mean force, absorbed energy, and CFE are higher. This combination is especially attractive for impact-energy absorbers, where a high transmitted peak load may be undesirable even when the total absorbed energy is large.

The results also demonstrate that the effect of functional grading is strongly topology dependent. The re-entrant architecture deforms primarily through rotation and bending of inclined cell walls followed by increasing ligament interaction and compaction. Introducing a gradient in parent-material strength directly modifies the resistance associated with these sequential mechanisms. The gradient therefore interacts with the intrinsic collapse kinematics of the re-entrant cells rather than acting as an independent material correction.

Because geometry, density, and mass were maintained constant, the differences between ISO and FGM responses cannot be attributed to changes in relative density or to additional structural material. This is a significant feature of the present comparison. Improvements in CFE and energy absorption are obtained through redistribution of existing material strength along the crushing direction rather than by increasing structural mass or altering the re-entrant geometry.

The adopted rate-independent material model further clarifies the physical origin of the observed velocity effects. No strain-rate-dependent increase in yield strength was introduced. Therefore, the changes in *F*_max_, *F_m_*, SEA, and CFE with impact velocity originate from structural dynamics. Inertia, collapse-front localization, progressive ligament rotation, contact, and densification determine how the same constitutive material resistance is mobilized at different loading velocities.

Overall, the results establish a clear difference between uniform and spatially graded parent-material strength in re-entrant honeycombs. The isotropic structure exhibits severe maximum forces at the lower investigated velocities, particularly when terminal compaction becomes dominant. Material grading suppresses these extreme loads while maintaining or increasing the useful crushing resistance. The improvement is particularly pronounced at 75 m/s, where the maximum force decreases by approximately 34.8%, the mean force and absorbed energy increase by about 11.5%, and CFE improves by approximately 70.8%.

At 100 m/s, both configurations show a transition toward a highly dynamic crushing regime characterized by increased mean resistance, stronger local oscillations, reduced terminal force amplification, and considerably higher CFE. Even under these conditions, the FGM structure maintains the more favorable performance, with greater mean crushing force and energy absorption combined with a lower maximum load.

These findings indicate that grading the yield resistance of the parent material along the crushing direction can be used to regulate the dynamic collapse of re-entrant auxetic structures without altering their geometry, mass, or relative density. The principal advantage of the approach is the redistribution of crushing resistance: relatively weak material assists controlled initiation of deformation, while progressively stronger material sustains resistance as collapse propagates through the network. The resulting response provides a combination of enhanced energy dissipation, reduced maximum transmitted force, and improved crush force efficiency, demonstrating the potential of material-property grading as a design strategy for re-entrant cellular structures subjected to dynamic in-plane loading.

## 4. Conclusions

The present study investigated the dynamic in-plane crushing response of isotropic and functionally graded re-entrant honeycomb structures subjected to different impact velocities. The main objective was to evaluate the influence of spatially varying parent-material strength while maintaining identical cellular geometry, relative density, and structural mass. The isotropic configuration employed a uniform yield strength, whereas the functionally graded model incorporated a continuous increase in yield strength from the impact side toward the opposite boundary. A rate-independent elastic–perfectly plastic material representation was adopted to isolate the contribution of structural inertia, progressive collapse, contact interactions, and deformation localization.

The results revealed that the proposed material-strength gradient significantly modifies the crushing behavior of the re-entrant topology. In comparison with the isotropic configuration, the functionally graded structure consistently reduced the maximum crushing force while maintaining or increasing the sustained crushing resistance. This behavior indicates that the grading strategy redistributes the resistance developed during deformation rather than simply reducing the overall strength of the cellular structure.

The absorbed energy and specific energy absorption also showed favorable trends for the graded configurations. The FGM structures generally dissipated more energy over the prescribed crushing displacement, particularly as the impact velocity increased. At the same time, crush force efficiency was improved, indicating a more favorable balance between the mean crushing resistance and the maximum transmitted load. The improvement became especially pronounced at the intermediate and higher impact velocities, where the interaction between the propagating collapse front and the material-strength gradient was more significant.

The observed response can be attributed to the spatial arrangement of plastic resistance. The lower-strength region near the impact boundary facilitates the initiation of localized deformation, whereas the progressively stronger material encountered during subsequent crushing provides increasing resistance to continued collapse. This mechanism promotes a more distributed crushing response and suppresses excessive force concentration during compaction.

Overall, the findings demonstrate that functional grading of the parent-material yield strength is an effective means of tailoring the dynamic behavior of re-entrant auxetic honeycombs without altering their geometry or mass. The proposed approach offers a promising route for improving load regulation, energy dissipation, and crushing efficiency in cellular structures intended for impact and crashworthiness applications.

## Data Availability

The original contributions presented in this study are included in the article. Further inquiries can be directed to the corresponding author.
